# Multiple roles for membrane-associated protein trafficking and signaling in gravitropism

**DOI:** 10.3389/fpls.2012.00274

**Published:** 2012-12-11

**Authors:** Allison K. Strohm, Katherine L. Baldwin, Patrick H. Masson

**Affiliations:** ^1^Laboratory of Genetics, University of Wisconsin-MadisonMadison, WI, USA; ^2^Graduate Program in Cellular and Molecular Biology, University of Wisconsin-MadisonMadison, WI, USA

**Keywords:** gravitropism, *Arabidopsis*, endomembrane, vacuole, trafficking, PIN, phosphatidylinositol, auxin transport

## Abstract

Gravitropism is a process that allows plant organs to guide their growth relative to the gravity vector. It requires them to sense changes in their orientation and generate a biochemical signal that they transmit to the tissues that drive organ curvature. Trafficking between the plasma membrane and endosomal compartments is important for all of these phases of the gravitropic response. The sedimentation of starch-filled organelles called amyloplasts plays a key role in sensing reorientation, and vacuolar integrity is required for amyloplast sedimentation in shoots. Other proteins associated with the vesicle trafficking pathway contribute to early gravity signal transduction independently of amyloplast sedimentation in both roots and hypocotyls. Phosphatidylinositol signaling, which starts at the plasma membrane and later affects the localization of auxin efflux facilitators, is a likely second messenger in the signal transduction phase of gravitropism. Finally, membrane-localized auxin influx and efflux facilitators contribute to a differential auxin gradient across the gravistimulated organs, which directs root curvature.

## INTRODUCTION TO GRAVITROPISM

Gravitropism is a dynamic process that involves the perception of an organ’s abnormal orientation within the gravity field, a transduction of the corresponding information into a biochemical signal, the transmission of this signal to a site of response, and organ curvature. Proper curvature therefore requires the coordination of multiple cellular activities including signal transduction, phytohormone transport, and cell expansion. Published work discussed in this review, mostly on *Arabidopsis*, indicates that protein trafficking through the endomembrane system plays a critical role in all of these processes.

Gravitropism begins with signal perception. In *Arabidopsis* roots, the specialized cells that sense gravity, or statocytes, are located in the root tips within the columella region of the cap (Blancaflor et al., 1998; Tsugeki and Fedoroff, 1999; Kiss, 2000); in shoots, the endodermis contains the statocytes (Fukaki et al., 1998). Both root columella and shoot endodermal cells contain dense, starch-filled amyloplasts that sediment to the lower sides of the statocytes upon gravistimulation (Caspar and Pickard, 1989; Kiss et al., 1989; Leitz et al., 2009). After amyloplast sedimentation, an auxin gradient is generated (part of the biochemical signal discussed above) and transmitted so that the auxin concentration on the lower side of the organ is higher than the concentration along its upper side (Ottenschlager et al., 2003). This typically promotes downward curvature of roots and upward curvature of shoots (Salisbury et al., 1988; Young et al., 1990).

The steps connecting amyloplast sedimentation and auxin redistribution in the signal transduction phase of gravitropism are still unclear, although several genes have been implicated in this phase. The molecular and functional analysis of some of these genes has suggested roles for endomembrane trafficking in this process. One possible model for signal perception involves the activation of stretch-activated mechanosensitive ion channels within membranes pressed upon by sedimenting amyloplasts (Leitz et al., 2009). Alternatively, in the ligand-receptor model, the activation of a transduction pathway occurs through productive interactions between sedimenting plastid-borne molecules and receptors associated with lower membranes (Braun, 2002). Lastly, in the hydrostatic pressure model, cellular machinery detects a pressure differential between the upper and lower sides of the statocytes caused by the weight of the entire protoplast on the cell wall (Staves, 1997). There is also substantial evidence for root gravity sensing outside of the columella cells that could involve an amyloplast-independent mechanism (Wolverton et al., 2002).

Researchers have proposed that several secondary messengers contribute to the signal transduction phase of gravitropism. For example, Ca^2^^+^ changes occur in response to gravistimulation, although studies have not found them in the columella cells (Plieth and Trewavas, 2002; Toyota et al., 2008). Cytosolic pH changes, however, do occur in the columella cells upon gravistimulation, and changing the pH alters the gravitropic response (Scott and Allen, 1999; Monshausen et al., 2011). Inositol 1,4,5-triphosphate (InsP_3_) also appears to contribute to the formation of the auxin gradient possibly through a role in vesicle trafficking (Perera et al., 1999; Wang et al., 2009).

In contrast to the signal perception phase of gravitropism, how a plant generates, maintains, and transmits the auxin gradient, as well as how this gradient dictates differential cell expansion, are better understood. The auxin efflux facilitators PIN-FORMED 3 (PIN3) and PIN7 show a distinct relocalization to the lower side of the root cap columella cells in response to gravistimulation that initiates the differential flow of auxin toward the lower flank of the root (Friml et al., 2002b; Kleine-Vehn et al., 2010). Other auxin transporters help to generate and propagate this gradient along the root, and protein trafficking is critical in this step. Auxin then may bind to one of two proposed auxin receptor classes, the AUXIN-BINDING PROTEIN 1 (ABP1) receptor or the TRANSPORT-INHIBITOR-RESISTANT 1 (TIR1)/AUXIN SIGNALING F-BOX (AFB) proteins. TIR1/AFB receptors bind auxin in a complex with an Aux/indole-3-acetic acid (IAA) regulatory protein, which is degraded upon auxin binding (Dharmasiri et al., 2005; Kepinski and Leyser, 2005). This de-represses auxin-response factors, which can then activate or suppress target genes to cause differential cell expansion on the upper and lower sides of roots and shoots. Although its mechanism of action is less clear, ABP1 is required for auxin responses at the plasma membrane and auxin-responsive gene expression changes, and it has been proposed to coordinate cell division and cell expansion (Shi and Yang, 2011). For more information on the overall gravitropic response, please see a recent review (Morita, 2010; Strohm et al., 2012).

## ENDOMEMBRANE SYSTEM COMPONENTS ARE IMPORTANT FOR GRAVITY PERCEPTION AND EARLY GRAVITY SIGNAL TRANSDUCTION

Endomembrane system components are required for normal shoot and root gravitropism in *Arabidopsis*. Endocytotic pathways mediate the transport of proteins from the plasma membrane in order to control their recycling via the endosome or their degradation. Many proteins targeted to vacuoles are transported from the ER, to the Golgi, and then to the vacuole, although a Golgi-independent pathway also exists. Furthermore, some endocytosed plasma membrane proteins are also targeted to the vacuole. Prevacuolar compartments (PVCs), also called multivesicular bodies (MVBs), mediate Golgi or plasma membrane to vacuole transport. For more information, see a recent review on this process (Reyes et al., 2011). Genetic screens for shoot gravitropism mutants revealed a contribution of vesicular trafficking to vacuoles in gravitropism. Similarly, a screen designed to find compounds that reduced hypocotyl gravitropic responses identified several small molecules that link gravitropism and endomembrane trafficking. Although characterization of the proteins that interact with these molecules is still underway, two of the compounds reduce gravitropic responses and disrupt the endomembrane system despite having no apparent effect on auxin, suggesting an auxin-independent role for endomembrane trafficking in gravitropism (Surpin et al., 2005).

### VACUOLAR INTEGRITY IS ESSENTIAL FOR AMYLOPLAST SEDIMENTATION IN SHOOTS

Four *shoot gravitropism* (*sgr*) mutants have been identified that share similar phenotypes and suggest a connection between vacuole integrity, amyloplast sedimentation, and shoot gravitropic responses. SGR3/VAM3 and SGR4/VTI11/ZIG are SNARES, which are named for SNAP (soluble NSF attachment protein) receptors and are small proteins that mediate vesicle fusion. They are divided into vesicle-SNAREs (v-SNAREs), which are located on vesicle membranes, and target-SNARES (t-SNAREs), which are located on target membranes. SGR3 is a t-SNARE (Sato et al., 1997), and SGR4 is a v-SNARE (Zheng et al., 1999). SGR8/GRV2/KAM2 is a DnaJ domain-containing peripheral membrane protein that localizes to late endosomes (Silady et al., 2004, 2008). Lastly, *SGR2* encodes a vacuole-localized protein homologous to the bovine testis phosphatidic acid-preferring phospholipase A1 (PA-PLA1; Kato et al., 2002).

#### *sgr2*, *sgr3*, *sgr4*, and *sgr8* share reduced shoot gravitropic responses, abnormal amyloplast localization, and altered vacuole structures

*sgr2*, *sgr3*, *sgr4*, and *sgr8 *mutants all exhibit strongly reduced shoot gravitropic responses but normal or slightly enhanced phototropic and root gravitropic responses; *sgr2* and *sgr4* mutants also display very slow hypocotyl gravitropism (Fukaki et al., 1996b; Yamauchi et al., 1997; Kato et al., 2002; Yano et al., 2003; Silady et al., 2004). All of these mutants show a generally intact tissue structure consisting of a single layer of epidermis, three to four layers of cortex, and one layer of endodermis, although the *sgr2*, *sgr4*, and *sgr8* mutants show some pleiotropic phenotypes including altered cell size and shape (Kato et al., 2002; Yano et al., 2003; Silady et al., 2004). This suggests that these genes are likely to function directly in gravitropism and do not simply have missing or disorganized statocytes.

In wild-type plants, amyloplasts in shoot endodermal cells are found sedimented on the lower sides of the cells (Morita et al., 2002). They are wrapped in thin, tunnel-like cytoplasmic layers surrounded by vacuolar membranes that are called transvacuolar strands, which pass through the vacuole and are connected to the peripheral cytoplasm. Amyloplasts can pass through these transvacuolar strands (Saito et al., 2005). However, in *sgr2*, *sgr3*, *sgr4*, and *sgr8* mutants, the endodermal amyloplasts are found throughout both the upper and lower sides of the cells where they localize outside of the vacuole (instead of within the transvacuolar strands), often pressed against the cell periphery (Morita et al., 2002; Yano et al., 2003; Silady et al., 2004). At least *sgr2* and *sgr4* amyloplasts can be stained with potassium iodide, suggesting that they do accumulate starch, although a few amyloplasts appeared to contain slightly less starch than wild-type (Morita et al., 2002). Together, these data suggest that altered amyloplast localization, rather than reduced starch accumulation, results in the abnormal gravitropic responses of these mutants.

*sgr2*, *sgr3*, *sgr4*, and *sgr8* also all show altered vacuolar phenotypes. *sgr2* and *sgr4* both have aberrant vacuolar components in the cytoplasm, although these compartments differ between mutants (Morita et al., 2002). *sgr3* vacuolar membranes form irregular curves and do not properly surround the amyloplasts (Yano et al., 2003). *sgr8 *mutants have irregularly shaped vacuoles and aggregates of endosomes, which suggests that they might not properly fuse the tonoplast and vesicular membranes (Silady et al., 2008).

#### Golgi-to-vacuole targeting is critical for proper amyloplast localization in shoots

*SGR2*, *SGR3*, *SGR4*, and *SGR8* are all expressed in all tissues examined, and at least for *SGR2*, *SGR3*, and *SGR4*, expression in the endodermis is sufficient to rescue the gravitropic defects of the mutants (Zheng et al., 1999; Morita et al., 2002; Yano et al., 2003; Silady et al., 2004). This indicates that these proteins’ contribution to gravitropism occurs within the statocytes. In root cells, SGR4 colocalizes with ELP, a vacuolar cargo receptor located on the trans-Golgi network, as well as with PEP12, a t-SNARE located at the PVC (Zheng et al., 1999). Experiments have shown that SGR4 can substitute for yeast Vti1p in vesicle transport from the Golgi to the PVC (Zheng et al., 1999). SGR3 localizes to the vacuole or the PVC, and coimmunoprecipitation experiments suggest that it forms a complex with SGR4 (Yano et al., 2003). Similarly, SGR8 plays a role in trafficking from the PVC to the tonoplast (Silady et al., 2008). Together, these data suggest that trafficking from the Golgi to the vacuole plays an important role in shoot and hypocotyl gravitropism, possibly by providing a cellular environment that is favorable to amyloplast sedimentation upon gravistimulation.

The putative phospholipase SGR2 also localizes to vacuolar membranes (Morita et al., 2002). It is therefore possible that SGR3, SGR4, or SGR8 directly mediates the localization of SGR2 or that another cargo protein transported by SGR3, SGR4, or SGR8 is important for the localization or activity of SGR2. Alternatively, SGR2 may contribute to gravitropism independently of SGR3, SGR4, and SGR8. While the exact function of SGR2 is still unclear, it is possible that it mediates the degradation of phospholipids that dictate the composition of membranes in order to modify their properties. This could consequently result in altered amyloplast sedimentation and slow gravitropic curvature. Another possibility is that cleavage of phospholipids by SGR2 creates signaling molecules required for gravitropism (Kato et al., 2002; Morita et al., 2002).

Unlike amyloplasts in shoots, those in root columella cells are not enveloped in vacuolar membranes and move through the cytoplasm instead of within transvacuolar strands (Zheng and Staehelin, 2001; Leitz et al., 2009). There is also no large central vacuole in columella cells like there is in shoot endodermal cells. Consistent with these observations, none of the mutations identified thus far as affecting root gravitropism have been associated with defects in vacuolar biogenesis or function.

### SOME ENDOMEMBRANE SYSTEM-ASSOCIATED PROTEINS MEDIATE EARLY GRAVITY SIGNAL TRANSDUCTION INDEPENDENTLY OF AMYLOPLAST SEDIMENTATION

*ALTERED RESPONSE TO GRAVITY 1* (*ARG1*/*RHG*) and its paralog *ARG1-LIKE 2* (*ARL2*/*GPS4*) encode DnaJ-domain-containing peripheral membrane proteins that are necessary for full root and hypocotyl gravitropism (Fukaki et al., 1997; Sedbrook et al., 1999; Boonsirichai et al., 2003; Guan et al., 2003; Luesse et al., 2010). GFP-ARG1 fusions localize to components of the vesicle trafficking pathway including the ER, the Golgi, and vesicles near the plasma membrane, as well as the cell plate. Additionally, upon treatment with brefeldin A (BFA), which disrupts vesicle trafficking, cMyc-ARG1 accumulates in BFA-induced compartments as do many proteins known to be associated with vesicle trafficking (Boonsirichai et al., 2003). ARG1 and ARL2 are required for the relocalization of PIN3 to the new lower sides of the columella cells upon gravistimulation, and at least ARG1 is required for the gravity-induced cytoplasmic alkalinization of the columella cells. Both of these processes are important in generating an auxin gradient (Boonsirichai et al., 2003; Harrison and Masson, 2008). These genes are especially interesting because *arg1* and *arl2* mutants display normal phototropism, amyloplast starch accumulation, amyloplast sedimentation, responses to phytohormones, and responses to auxin transport inhibitors (Fukaki et al., 1997; Sedbrook et al., 1999; Guan et al., 2003; Stanga et al., 2009). Although the specific molecular function of ARG1 and ARL2 remains unclear, these data suggest that they play a role in the early gravity signal transduction steps that connect amyloplast sedimentation and auxin redistribution.

## PHOSPHATIDYLINOSITOL SIGNALING MEDIATES VESICLE TRAFFICKING, AUXIN GRADIENT FORMATION, AND THE GRAVITROPIC RESPONSE

Phosphatidylinositol monophosphate 5-kinase (PIP5K) catalyzes the synthesis of phosphatidylinositol 4,5-bisphosphate (PIP_2_), a plasma membrane-localized phospholipid. PIP_2_ is then cleaved by phospholipase C (PLC) to produce the second messenger InsP_3_, which diffuses throughout the cell, and diacylglycerol (DAG), which stays in the membrane. Inositol polyphosphate 5-phosphatases (InsP 5-ptases) dephosphorylate InsP_3_ to stop its activity. In animals, InsP_3_ can trigger Ca^2^^+^ release from the ER and the vacuole, and Ca^2^^+^ itself is another possible second messenger in gravity signal transduction in plants (Plieth and Trewavas, 2002; Monshausen et al., 2011). Additional research in both animals and plants has shown that PIP_2_ can bind actin-interacting enzymes, endocytic and exocytic-related proteins, ion channels, and regulators of vesicle trafficking. Please see the following reviews for additional information (Wasteneys and Galway, 2003; Haucke, 2005).

### InsP_3_ MAY ACT AS A SECOND MESSENGER IN GRAVITROPISM SIGNALING

Multiple lines of evidence point to a role for phosphatidylinositol signaling in gravity signal transduction. InsP_3_ levels increase threefold on both the upper and lower sides of gravistimulated oat pulvini after only 15 s. Over the next 30 min, InsP_3_ fluxes continue, resulting in a threefold increase in the levels on the upper compared to the lower side. After about an hour, InsP_3_ returns to its basal level (Perera et al., 2001). Several other observations also support a role for InsP_3_ in gravitropism. Phosphatidylinositol 4-phosphate 5-kinase levels increase in the lower halves of gravistimulated pulvini, suggesting that PIP_2_ biosynthesis increases in this region (Perera et al., 1999). Additionally, inhibiting PLC also blocks the long-term InsP_3_ increase and reduces gravitropic bending (Perera et al., 2001). Some genes show InsP_3_-dependent changes in expression in response to gravitropic and/or phototropic stimuli, suggesting that this second messenger may play a key role in coordinating these two responses (Salinas-Mondragon et al., 2010).

*Arabidopsis* inflorescence stems can perceive a change in orientation while at 4°C but cannot respond until after they are returned to room temperature (Fukaki et al., 1996a). InsP_3_ changes are similar in plants gravistimulated at 4°C and at room temperature, and plants expressing a constitutively active InsP 5-ptase show decreased bending after gravistimulation at 4°C and a subsequent return to room temperature (Perera et al., 2001, 2006). These results support the hypothesis that phosphatidylinositol signaling functions early in gravity signal transduction.

### PLANTS CARRYING MUTATIONS IN GENES ASSOCIATED WITH PHOSPHATIDYLINOSITOL SIGNALING SHOW ALTERED GRAVITROPIC AND AUXIN-RELATED PHENOTYPES

PIP5K and InsP 5-ptases are each encoded by 15 genes in *Arabidopsis*. *pip5k2* seedlings have decreased PIP_2_ levels, and *5-ptase13* mutants are likely to have a decreased ability to dephosphorylate InsP_3_ (Wang et al., 2009; Mei et al., 2012). Therefore, it is not surprising that these mutants share many opposite phenotypes. *pip5k2* seedlings respond slowly to gravity, while *5-ptase13* mutants show an enhanced response (Wang et al., 2009; Mei et al., 2012). In agreement with this finding, plants expressing a constitutively active InsP 5-ptase do not exhibit the characteristic InsP_3_ increase in response to gravistimulation and show decreased gravitropic bending (Perera et al., 2006). *pip5k2* mutants are more sensitive to the polar auxin transport inhibitor 1-N-naphthylphthalamic acid (NPA) than are wild-type plants, which suggests impaired polar auxin transport in this mutant (Mei et al., 2012). In contrast, *5-ptase13* mutants show a reduced response to NPA, which indicates increased polar auxin transport (Wang et al., 2009). Plants carrying the constitutively active InsP 5-ptase also show decreased basipetal auxin transport (Perera et al., 2006). Indeed, a greater percentage of *5-ptase13* mutants and a smaller percentage of *pip5k2* mutants generate an asymmetric auxin gradient in roots in response to gravistimulation compared to wild-type seedlings, resulting in altered gravitropic phenotypes (Wang et al., 2009; Mei et al., 2012).

### PHOSPHATIDYLINOSITOL SIGNALING AFFECTS VESICLE TRAFFICKING AND PIN PROTEIN TURNOVER

Phosphatidylinositol signaling is required for proper vesicle trafficking that leads to the establishment of an auxin gradient. PIN auxin efflux facilitators play important roles in controlling the direction and rate of auxin fluxes that allow for differential cell expansion upon gravistimulation (see The PIN Family of Auxin Efflux Facilitators). Normally PIN proteins cycle between the plasma membrane and endosomal compartments. This process is sensitive to BFA and requires clathrin-mediated endocytosis (Steinmann et al., 1999; Friml et al., 2002b; Geldner et al., 2003; Dhonukshe et al., 2007; Kleine-Vehn et al., 2010). Compared to wild-type, *5-ptase13* mutants have an increased ability to internalize the endocytosis marker FM4-64, are less sensitive to BFA, and show faster resumption of PIN1 and PIN2 polar localization at the plasma membrane after BFA removal (Wang et al., 2009). In contrast, *pip5k2* mutants show a decreased ability to internalize FM4-64, increased sensitivity to BFA, slower recovery after BFA removal, and decreased cycling of PIN2 and PIN3 (Mei et al., 2012). The phosphatidylinositol-3-kinase (PI3K) inhibitor wortmannin also results in altered PIN protein localization and gravitropic defects (Jaillais et al., 2006; Kleine-Vehn et al., 2008). Together, these data indicate a role for phosphatidylinositol signaling in vesicle trafficking that affects PIN protein turnover and the generation of the auxin gradient that is required for differential cell elongation in response to a gravity stimulus. It remains possible, however, that the *5-ptase13* and *pip5k2* mutants have altered membrane lipid composition, which is known to affect PIN cycling (Men et al., 2008).

## AUXIN TRANSPORT ACROSS CELL MEMBRANES RESULTS IN AN AUXIN GRADIENT THAT DIRECTS DIFFERENTIAL CELL EXPANSION

**FIGURE 1:**
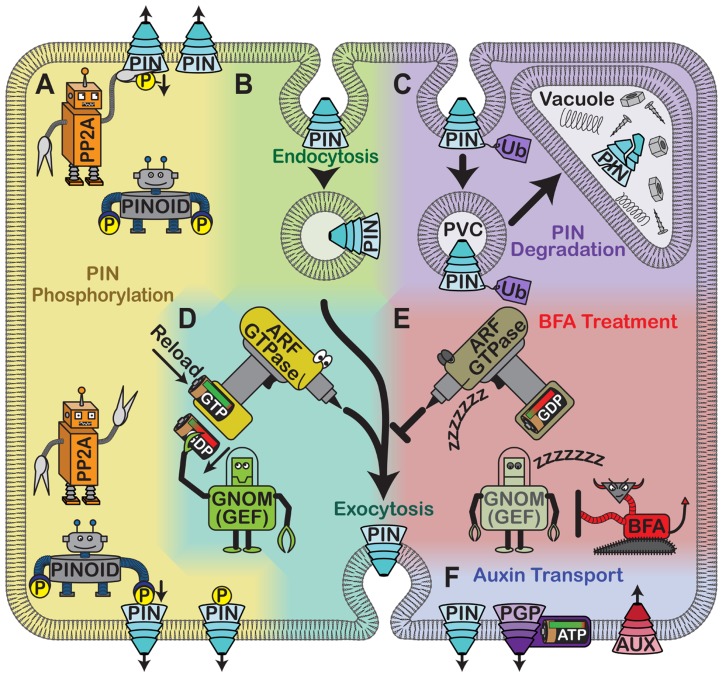
**Cellular control of auxin carriers**. **(A)** Phosphorylation by PINOID kinase and dephosphorylation by PP2A regulate PIN protein localization. **(B)** PIN proteins are removed from the plasma membrane through clathrin-mediated endocytosis into endocytic compartments. **(C)** PIN proteins may also be ubiquitylated and targeted to the vacuole via PVCs for degradation. **(D)** Alternatively, following endocytosis PIN proteins may be exocytosed in a selective, polar manner that requires the activity of an unidentified ARF GTPase. The activity of the GTPase is controlled by a GEF called GNOM, which removes the used GDP and allows fresh GTP to reload. **(E)** Treating plants with BFA inhibits GNOM, which likely inactivates the ARF GTPase. As a result, PIN proteins accumulate in intracellular aggregates termed BFA bodies. **(F)** Auxin can be actively transported across the plasma membrane. PIN proteins are gradient-powered auxin efflux carriers. Members of the ABC transporter family are ATP-driven and act as either auxin influx or efflux facilitators. AUX1 and its relative LAX are auxin influx carriers that use an existing ion gradient to allow auxin into cells.

The major natural auxin, IAA, is a weak acid that can diffuse through membranes only when protonated (Rubery and Sheldrake, 1974; Raven, 1975). The pH in the apoplast remains low, and so a proportion of IAA molecules are protonated, which allows them to diffuse across the membrane into the cytoplasm (Rubery and Sheldrake, 1974; Raven, 1975). Additional apoplastic IAA can be actively imported by the AUX1 and LIKE-AUX1 (LAX) permeases and at least one ATP-binding cassette (ABC) transporter/P-glycoprotein (PGP) (Marchant et al., 1999; Santelia et al., 2005; Yang et al., 2006; Yang and Murphy, 2009; Péret et al., 2012). Once in the cytoplasm, far fewer IAA molecules are protonated, being exposed to more neutral pH, and active auxin efflux mediated by PIN proteins and some ABC transporters is required (**Figure 1F**). The polar localization of these proteins at the plasma membrane can dictate directional auxin transport within cell files (Wisniewska et al., 2006). In vertically growing roots, this results in the flow of auxin down the vasculature to the columella cells where it is redirected along the outer layers of the root in what is termed the reverse fountain model of auxin transport (Swarup and Bennett, 2003). Upon gravistimulation, the PIN3 and PIN7 auxin efflux carriers switch from a non-polar localization to a preferential distribution at the lower side of the plasma membrane (Friml et al., 2002b; Kleine-Vehn et al., 2010). This causes auxin to accumulate in the lower sides of shoots and roots where it alters cell expansion rates to cause organ curvature (Salisbury et al., 1988; Young et al., 1990). Vesicle trafficking therefore plays a critical role in mediating this auxin gradient through its effects on the abundance, activity, and subcellular localization of auxin efflux and influx carriers. Membrane composition and differences in the sensitivity of certain cells to auxin over time also influence curvature kinetics (Willemsen et al., 2003; Benjamins and Scheres, 2008).

### THE PIN FAMILY OF AUXIN EFFLUX FACILITATORS

There are eight PIN proteins in *Arabidopsis*, and at least five of them function directly or indirectly in gravitropism. This is achieved through their asymmetric localization at the plasma membrane, which can determine the direction of auxin flow (Wisniewska et al., 2006). These proteins often have overlapping functions; when one protein is non-functional, auxin-dependent ectopic expression of other PIN proteins can sometimes compensate for the loss (Blilou et al., 2005; Vieten et al., 2005).

#### PIN proteins are required for the generation and propagation of the gravity-induced auxin gradient

PIN1 localizes to the rootward sides of the cells that form the vasculature whereas PIN4 localizes to the rootward sides of the proximal meristem cells; the latter also shows non-polar localization in the columella cells (Friml et al., 2002a). These patterns suggest that PIN1 and PIN4 play indirect roles in gravitropism by contributing to auxin efflux through the vasculature to the columella cells (Gälweiler et al., 1998; Geldner et al., 2001; Friml et al., 2002a). This is important for auxin to be transported to the root tip so that it can later be distributed laterally across the cap and up to the elongation zones upon gravistimulation. While mutations in *PIN4* cause root meristem disorganization that makes it difficult to analyze their gravitropic responses, *pin1* mutants have normal roots with no gravitropic defects, suggesting that other *PIN*s are able to compensate for the loss of this gene (Friml et al., 2002a).

In contrast, PIN3 and PIN7 may function immediately upon gravistimulation to generate the initial auxin gradient across the cap. PIN3 is normally expressed in the upper S1 and S2 layers of the columella cells, while PIN7 localizes to the S2 and S3 tiers. However, PIN7 expands its expression into the S1 layer in *pin3* mutants, suggesting its ability to compensate for the loss of PIN3 (Kleine-Vehn et al., 2010). In roots growing vertically, these proteins show a generally non-polar localization in the columella cells, but upon gravistimulation they are internalized and resorted into vesicles that direct them to the lower plasma membrane. This gravity-induced relocalization of the PIN3 and PIN7 proteins within the statocytes may be responsible for the development of a lateral auxin gradient across the cap, with accumulation on the new lower side of the root (Friml et al., 2002b; Kleine-Vehn et al., 2010). Consistent with this conclusion, the *pin3* and *pin7* mutants show gravitropism defects, and the *pin3 pin7 *double mutant shows stronger defects than either single mutant (Friml et al., 2002b; Kleine-Vehn et al., 2010).

PIN2/EIR1/AGR1/WAV6 localizes to the shootward sides of lateral root cap and epidermal cells where it plays a critical role in transporting auxin from the cap to the elongation zone both in vertically growing roots and upon gravistimulation. It also localizes to the rootward sides of the cortical cells in the meristem, where it may play a negative regulatory role that allows for optimal auxin fluxes in this region (Müller et al., 1998; Blilou et al., 2005; Abas et al., 2006; Rahman et al., 2010). Here it may also contribute to an auxin reflux loop through the root epidermal and cortical cells in which the auxin maximum that forms on the lower side of the root is reinforced. *pin2* mutants do not establish an auxin gradient upon gravistimulation and therefore exhibit gravitropic defects (Luschnig et al., 1998; Müller et al., 1998; Abas et al., 2006).

#### PIN protein regulation affects gravitropic responses

PIN proteins can be regulated at the levels of transcription, protein stability, subcellular localization, and transport activity (Petrásek and Friml, 2009). Because auxin efflux requires a membrane H^+^ gradient and because PIN proteins do not have recognizable ATP-binding motifs, PIN proteins are thought to be gradient-driven secondary transporters (Krecek et al., 2009). However, they can act together with ATP-dependent ABC transporters when needed (Blakeslee et al., 2007).

***Guanine nucleotide exchange factors regulate PIN protein localization***. Intracellular trafficking is required for the polar localization of PIN proteins, which cycle between the plasma membrane and endosomal compartments (Steinmann et al., 1999; **Figure 1B**). GNOM is a GTP–GDP exchange factor (GEF) for ADP ribosylation factor (ARF) small G-proteins, which are important for cargo selection and vesicle budding (**Figure 1D**). BFA binds to ARF-GEF/ARF-GDP complexes and prevents ARF activation (Peyroche et al., 1999; Robineau et al., 2000). When this happens, PIN1 and PIN3 endocytosis continues, but these proteins are no longer secreted, causing them to accumulate in intracellular compartments (Geldner et al., 2001; Kleine-Vehn et al., 2010; **Figure 1E**). Therefore, BFA treatment blocks the relocalization of PIN3 to the lower membrane upon reorientation, alters auxin transport, and reduces gravitropism (Geldner et al., 2001; Kleine-Vehn et al., 2010; Rahman et al., 2010). Plants expressing a BFA-resistant version of GNOM, however, show proper PIN1 and PIN3 localization, robust auxin gradients after gravistimulation, and normal gravitropism even in the presence of BFA (Geldner et al., 2003; Kleine-Vehn et al., 2010). This confirms that GNOM regulates PIN1 and PIN3 localization and demonstrates that ARF-GEFs can modulate certain endosomal trafficking routes. BFA also partially affects PIN2 localization, suggesting that the gravitropic defects associated with BFA treatment might not be due entirely to its effects on PIN1 and PIN3 (Geldner et al., 2003).

SPIKE1 (SPK1) acts as a GEF for Rho-like GTPase from Plants 6 (ROP6; Basu et al., 2008). In *spk1* mutants, PIN2 levels at the plasma membrane are decreased. Consistent with this, *spk1* mutants show a less robust auxin gradient upon reorientation and a slow gravitropic response (Lin et al., 2012). Plants carrying mutations in *ROP6 *or its effector *ROP-INTERACTIVE CRIB MOTIF 1 *(*RIC1*) share some of these phenotypes, whereas ROP6 overexpression causes an increased gravitropic response (Chen et al., 2012; Lin et al., 2012). Normally auxin increases active ROP6 levels, but this does not happen in *spk1* mutants. Additionally, while exogenous application of the synthetic auxin 1-naphthalene acetic acid (1-NAA) normally prevents BFA-induced PIN2 accumulation in internal BFA compartments, *spk1*, *rop6*, and *ric1* mutants do not show this effect (Lin et al., 2012). These data suggest that SPK1, ROP6, and RIC1 inhibit PIN2 internalization through their effects on auxin signaling.

***Protein degradation, protein phosphorylation, and small peptides also regulate PIN2 localization***. PIN2 is also clearly regulated at the protein stability level. Upon gravistimulation, PIN2 is internalized and degraded preferentially on the upper side of the root, which is required for the generation of the auxin gradient. When BFA or the proteasome inhibitor MG132 is applied, this asymmetry is disrupted and PIN2 levels increase; this correlates with gravitropism defects (Abas et al., 2006). PIN2 endocytosis and targeting to the vacuole are normally triggered by ubiquitylation (**Figure 1C**). However, in *pin2* mutants in which six or more potential ubiquitylation sites are mutated, PIN2 is not internalized and targeted to the vacuole upon gravistimulation. Therefore, PIN2 levels stay constant at the plasma membrane in these mutants, and these seedlings do not form a robust auxin gradient upon reorientation (Leitner et al., 2012). Short-term auxin treatment also interferes with intracellular PIN2 accumulation, but long-term treatment causes PIN2 internalization and degradation (Abas et al., 2006). This may reflect a feedback mechanism in which PIN2 is degraded after the auxin level reaches a threshold, preventing additional auxin transport and excessive root curvature. Ubiquitylation could control the rate of PIN2 degradation in this process.

BFA inhibits the targeting of PIN2 to the vacuole, which suggests the involvement of an ARF-GEF. However, plants expressing the BFA-resistant GNOM showed BFA-sensitive PIN2 vacuolar targeting, indicating that the ARF-GEF of interest is not GNOM. Like GNOM, SORTING NEXIN 1 (SNX1) localizes to endosomal compartments and is BFA-sensitive; however only SNX1 is sensitive to the PI3K inhibitor wortmannin (Jaillais et al., 2006; Kleine-Vehn et al., 2008). *snx1* mutants resemble weak allele *gnom* mutants, and *snx1 gnom* double mutants show enhanced abnormal phenotypes compared to the single mutants (Jaillais et al., 2006). This suggests that these genes function in different pathways but contribute to some of the same developmental processes. Upon wortmannin-treatment, PIN2 and SNX1 colocalize in compartments, and PIN2 levels at the plasma membrane are reduced in *snx1* mutants (Jaillais et al., 2006; Kleine-Vehn et al., 2008). However, SNX1 does not appear to directly mediate the localization or recycling of PIN2 (Kleine-Vehn et al., 2008). Instead, wortmannin likely causes PIN protein mislocalization through its interference with sorting between the PVC and the Golgi (Matsuoka et al., 1995). Proper PIN2 localization depends on its targeting to the vacuole where it is degraded, and wortmannin blocks this (Jaillais et al., 2006; Kleine-Vehn et al., 2008). Accordingly, long-term wortmannin treatment results in phenotypes reminiscent of altered auxin transport, including defective root and hypocotyl gravitropism (Jaillais et al., 2006). SNX1 may therefore contribute to a feedback mechanism involved in PIN2 retrieval for recycling through its ability to mediate PIN2 translocation from the PVC to the vacuole.

PIN protein localization also depends on its phosphorylation state, which is mediated in part by the serine-threonine kinase PINOID (PID) and type 2A protein phosphatase (PP2A), which act antagonistically (Michniewicz et al., 2007 **Figure 1A**). PP2A subunits are encoded by multiple genes including *ROOTS CURL IN NPA 1 *(*RCN1*). Plants that overexpress PID, *rcn1 *mutants, and wild-type plants treated with the phosphatase inhibitor cantharidin all show increased shootward auxin transport, delayed auxin gradient formation upon gravistimulation, and randomized root growth; these phenotypes are rescued by blocking polar auxin transport (Christensen et al., 2000; Benjamins et al., 2001; Rashotte et al., 2001). The elevated auxin transport in these plants probably leads to auxin depletion in the root meristem, which prevents auxin gradient formation (Benjamins et al., 2001; Rashotte et al., 2001). This increased auxin transport is attributed to a rootward to shootward shift in the localization of some PIN proteins (Friml et al., 2004). PINOID and RCN1 partially colocalize with PIN proteins and mediate the phosphorylation states of their central hydrophilic loops (Michniewicz et al., 2007). This means that they can affect PIN2-mediated auxin fluxes upon gravistimulation (Shin et al., 2005). More specifically, PP2A and a PINOID kinase family member are known to mediate the polar targeting of PIN2 in meristematic cortical cells, which is necessary for a full gravitropic response (Rahman et al., 2010). These experiments show that the phosphorylation status of PIN proteins affects their localizations and in turn their abilities to regulate gravitropism.

In addition to intracellular trafficking, protein degradation, and phosphorylation, a recent study suggests that small secretory peptides can also regulate PIN protein localization and affect gravitropism. *GOLVEN* (*GLV*) genes encode these peptides, and overexpression or knockdown of these genes generally results in reduced root and hypocotyl gravitropism. Treatment with some of these peptides, which act locally, also correlates with reduced auxin gradient formation upon reorientation and results in gravitropic defects. *pin2* mutants are resistant to GLV peptide treatment, and PIN2 levels increase in the membrane fractions of wild-type plants treated with GLV peptides (Whitford et al., 2012). Therefore, it is thought that the GLV peptides, along with auxin, mediate PIN2 trafficking in order to generate the auxin gradient necessary for root curvature.

#### PIN proteins may promote growth in the organ curvature phase of gravitropism

Auxin can inhibit the internalization of many PIN proteins and prevent their constitutive cycling. This results in increased levels of PIN proteins at the plasma membrane, and so auxin stimulates its own efflux from cells. After gravistimulation, the inhibition of endocytosis corresponds with the formation of the auxin gradient (Paciorek et al., 2005). Therefore, the increased level of plasma membrane-associated PIN2 on the lower flank of gravistimulated roots may further enhance the auxin gradient.

Auxin also triggers cell wall loosening that is necessary for cell elongation during root curvature. In *Arabidopsis*, PIN1 mediates local auxin accumulation, and its polar localization corresponds to the direction of mechanical stress in shoot apices (Heisler et al., 2010). Work done in tomatoes shows that as tissue becomes more strained during growth, PIN1 shows an increase in overall abundance and a preferential localization at the plasma membrane. This contributes to auxin accumulation, which then promotes growth in a feed-forward loop. One possible mechanism for this is that local cell wall strain increases plasma membrane tension, which promotes exocytosis and blocks endocytosis. This could increase the amount of membrane-localized PIN1 relative to cytoplasmically-localized PIN1, although more complex models are also possible (Nakayama et al., 2012). It is possible that a similar process takes place upon gravistimulation, although this has not yet been addressed experimentally. For example, tissue strain during curvature could increase plasma membrane-localized PIN2 levels on the lower side of the root. This would increase the auxin concentration in this region and further inhibit curvature in a feed-forward manner.

### THE ABC TRANSPORTER FAMILY OF AUXIN EFFLUX AND INFLUX FACILITATORS

Members of the family of ATP-binding cassette (ABC) transporters couple ATP hydrolysis with the import and export of molecules such as xenobiotics, ions, sugars, lipids, peptides, and hormones including auxin across cell membranes. There are several lines of evidence that these proteins play critical roles in maintaining the auxin gradient that results in gravitropism.

#### ABC transporters regulate auxin fluxes

Multiple pieces of evidence support a role for several ABC-type transporters in auxin transport. First, the *Arabidopsis* PGP19/MDR1/ABCB19 protein and its closest relative ABCB1 directly act as auxin transporters when expressed in mammalian and yeast cells as well as in protoplast assays (Geisler et al., 2005; Yang and Murphy, 2009). Furthermore, *abcb19 *single mutants and to a greater extent *abcb19 abcb1* double mutants show decreased rootward auxin transport (Noh et al., 2001; Lewis et al., 2007). Similarly, plants carrying mutations in *ABCB4/PGP4/MDR4*, another ABC-type transporter with sequence similarity to *ABCB1* and *ABCB19*, show decreased shootward auxin transport (Santelia et al., 2005; Terasaka et al., 2005; Lewis et al., 2007).

Interestingly, ABCB1 shows a distinct polar localization in different cell types at the upper edge of the distal elongation zone. In the endodermal cells its localization is always shootward, and in the cortical cells it is most often shootward (Geisler et al., 2005). A similar result was found for ABCB19 (Blakeslee et al., 2007). On the other hand, ABCB4 shows rootward localization in the epidermal cells at the upper edge of the distal elongation zone while displaying apolar localization in S3 columella and adjacent root cap cells (Terasaka et al., 2005). These distinct localization patterns may help generate differential levels of auxin accumulation in different cells.

A phenotypic analysis of these mutants is also compatible with a role for these proteins in auxin transport. Indeed, *abcb19 *and *abcb19 abcb1 *mutants show epinastic, or downward-folding, cotyledons and first true leaves as do wild-type plants when treated with exogenous auxin (Noh et al., 2001). This is likely due to the improper accumulation of auxin in the cotyledons. These mutants also show increased sensitivity to 1-NAA, decreased sensitivity to NPA, and decreased auxin-responsive DR5::GUS expression (Geisler et al., 2005; Lin and Wang, 2005).

Together, these studies strongly suggest that these transporters help maintain proper auxin flow patterns, and additional work has shown that interactions between ABC transporters and other proteins play important roles in this process. Genetic interactions have been observed between *ABCB*s and *PIN*s, and coimmunoprecipitation and yeast two-hybrid experiments have shown that both ABCB1 and ABCB19 interact with PIN1 (Blakeslee et al., 2007). Additionally, *abcb19* and especially *abcb19 abcb1* mutants show diffuse, punctate, and discontinuous PIN1 localization, which is likely to result in randomized directions of auxin efflux (Noh et al., 2003). Heterologous coexpression studies have also shown that the rate of auxin transport is increased when these proteins colocalize compared to when only one of them is present. In contrast, when PIN2 and either ABCB1, ABCB4, or ABCB19 are coexpressed in HeLa cells, IAA efflux decreases when compared to when only one protein is expressed (Blakeslee et al., 2007). AGC kinases also mediate both PIN protein polarity and the auxin efflux activity of ABCB1 and ABCB19, suggesting that they regulate crosstalk between these auxin transporters (Christie et al., 2011; Henrichs et al., 2012). From these experiments, it appears that the ABC transporters and PIN proteins function separately but synergistically to provide both the specificity and the high rate of long-distance auxin transport.

Additionally, the immunophilin-like integral membrane protein required for brassinosteroid perception or signaling, TWISTED DWARF 1, interacts with both ABCB1 and ABCB19 (Geisler et al., 2003). *twd1* mutants exhibit epinastic cotyledons and a strong reduction in polar auxin transport like *abcb1 abcb19 * double mutants, suggesting that ABCB1 and ABCB19 form a complex with TWD1 (Geisler et al., 2003). It is possible that TWD1 regulates the transport activity of ABCB1 and ABCB19 or that it mediates ABCB–PIN interactions.

#### ABC transporters are required for normal gravitropic responses

Several experiments show that ABC transporters function in the auxin transport phase of gravitropism. Interestingly, *abcb19* hypocotyls respond to gravistimulation twice as quickly as wild-type plants, and they also exhibit an enhanced phototropic response (Noh et al., 2003). Similarly, the *abcb4* mutant shows a faster root gravitropic response than wild-type plants (Lewis et al., 2007). Experiments using the auxin-responsive DR5::GUS construct showed that these mutants form a more robust asymmetric auxin gradient across the root tip than wild-type plants (Lin and Wang, 2005; Lewis et al., 2007). The altered auxin efflux may therefore result in a steeper, although transient, auxin gradient upon gravistimulation. One possible explanation for this comes from studies showing that *PIN2* mRNA levels decrease with distance from the root tip, while *ABCB4* mRNA levels increase (Birnbaum et al., 2003). If this correlates with their contributions to auxin transport, the reduced shootward auxin transport as a result of the loss of *ABCB19* or *ABCB4* may cause auxin buildup in the elongation zone where it leads to an enhanced curvature response (Lewis et al., 2007). Surprisingly, despite the large reduction in rootward auxin transport, root gravitropic responses of *abcb19* mutants are normal (Lewis et al., 2007). This could be due to compensation by other ABC transporters or PIN proteins.

A screen for compounds that reduce hypocotyl gravitropic responses identified a molecule called Gravacin that also causes decreased auxin sensitivity, decreased auxin transport, and endomembrane system defects (Surpin et al., 2005; Rojas-Pierce et al., 2007). Subsequent work showed that *abcb19* and *twd1*, but not *abcb1*, are resistant to Gravacin (Rojas-Pierce et al., 2007). Gravacin targets *ABCB19* and disrupts the ABCB19–PIN1 complexes, thereby interfering with their auxin transport activity (Rojas-Pierce et al., 2007). Using Gravacin to perturb ABCB19 but not PIN proteins may be useful in further characterizing the role of ABC transporters in auxin fluxes and gravitropism.

### THE AUX AND LAX FAMILY OF AUXIN IMPORT CARRIER PROTEINS

In addition to auxin efflux, auxin flow into cells also contributes to the auxin gradient. While auxin influx can occur by diffusion, the auxin influx carriers AUX1 and LAX can also actively import IAA (Marchant et al., 1999; Yang et al., 2006; Yang and Murphy, 2009; Péret et al., 2012). Active auxin influx into particular cells might maintain proper auxin fluxes by counteracting auxin diffusion into other cells. *aux1*, but not *lax*, mutants are agravitropic, suggesting functional specialization within this gene family (Bennett et al., 1996; Péret et al., 2012). Because *aux1 *mutants are defective in active auxin uptake, they are therefore resistant to exogenous IAA and the auxin 2,4-dichlorophenoxyacetic acid (2,4-D), but not 1-NAA, which can diffuse easily through membranes (Maher and Martindale, 1980; Bennett et al., 1996). Similarly, 1-NAA, but not 2,4-D, rescues the *aux1* agravitropic root phenotype (Marchant et al., 1999). It is likely that 1-NAA is taken up by the root and redirected by an auxin efflux facilitator such as PIN2, which is expressed in the cortical and epidermal root tip cells like *AUX1 *(Müller et al., 1998; Marchant et al., 1999).

AUX1 functions in the signal transmission and curvature response phases, not the perception phase, of gravitropism. This is suggested by its expression in the regions of the root that respond to gravity (Marchant et al., 1999). Consistent with this result, AUX1 expression in only the lateral root cap and epidermal cells is sufficient to rescue the *aux1 *agravitropic phenotype (Swarup et al., 2005). This suggests that AUX1 contributes to gravitropism by facilitating shootward auxin transport from the root cap to the elongation zone (Swarup et al., 2005).

AUX1 also affects pH changes upon gravistimulation, suggesting a relationship between pH and auxin in gravitropism. Shortly after reorientation, wild-type roots show a decrease in pH on the upper side of the extracellular root surface and an increase on the lower side; this gradient occurs in the root cap as well as throughout the elongation zone (Monshausen et al., 2011). It might contribute to cell wall loosening to allow for cell expansion or even to signal transmission itself. *aux1* mutants, however, do not show this pH change. In fact, even when growing vertically, *aux1* mutant extracellular root surfaces are uniformly acidic instead of showing dynamic pH fluctuations like wild-type roots. Both the pH gradient and the pH dynamics are rescued by introducing AUX1 into only the lateral root cap and epidermal cells (Monshausen et al., 2011). It is possible that the pH gradient contributes to feedback mechanisms that regulate the gravity response by affecting AUX1-mediated auxin uptake.

## CONCLUSION

**FIGURE 2:**
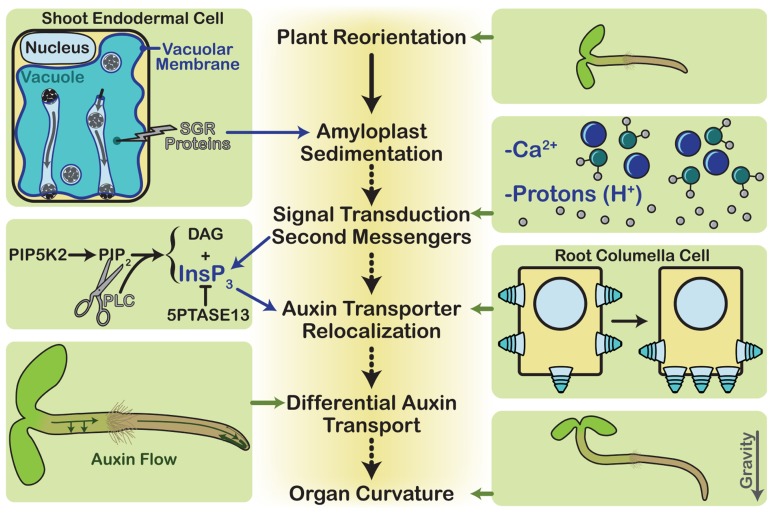
**Gravitropism overview**. The steps of gravitropism are shown down the center core of the diagram. During plant reorientation, a plant is rotated relative to the gravity vector. This results in the sedimentation of dense amyloplasts within the statocytes. In roots the statocytes are the columella cells, whereas in stems they are the endodermal cells. Each endodermal cell contains a large vacuole, and the amyloplasts must traverse it by tunneling through transvacuolar strands in order to reach the new lower side of the cell. This requires proper vacuole structure, which the SGR proteins mediate. Amyloplast sedimentation is then thought to activate signal transduction through second messengers, possibly calcium ions or protons. Another second messenger is InsP_3_, which is produced by cleavage of the phospholipid, PIP_2_. In a process that is not completely understood, the second messengers activate the relocalization of auxin transporters, such as PIN3 and PIN7 in the columella cells. The new polarized distribution of these auxin efflux carriers changes the flow of auxin throughout the plant. This differential auxin transport affects cell elongation rates, thereby resulting in organ curvature as the plant grows.

Upon gravistimulation, amyloplasts sediment to the lower sides of the statocytes. In endodermal cells, SGR proteins play a key role in this process by maintaining vacuolar membrane integrity. The amyloplasts then trigger a signal transduction cascade that may involve protons, calcium, and phosphatidylinositol signaling, which begins at the plasma membrane. Phosphatidylinositol signaling affects the cycling of auxin transporters, and changes in their localization at the plasma membrane cause auxin to accumulate in the lower side of the root and shoot. Here it affects cell elongation and causes the plant to realign itself with the gravity vector (**Figure 2**).

Therefore, through their roles in amyloplast sedimentation, phosphatidylinositol signaling, and auxin carrier localization, membranes contribute in multiple ways to all phases of gravitropism. The evolution of these complex processes allows plants to adapt to changing environments and to integrate their responses to gravity with those to a wide variety of other stimuli including touch and moisture gradients. Future work in this area will continue to clarify how membrane-associated signaling and trafficking contribute to gravitropism and other areas of plant growth and development.

## Conflict of Interest Statement

The authors declare that the research was conducted in the absence of any commercial or financial relationships that could be construed as a potential conflict of interest.
